# Effect of antibiotic-induced intestinal dysbacteriosis on bronchopulmonary dysplasia and related mechanisms

**DOI:** 10.1186/s12967-021-02794-6

**Published:** 2021-04-16

**Authors:** Xiao Ran, Yu He, Qing Ai, Yuan Shi

**Affiliations:** 1 Department of Neonatology, Children’s Hospital of Chongqing Medical University, National Clinical Research Center for Child Health and Disorders; Ministry of Education Key Laboratory of Child Development and Disorders, No.136 Zhongshan 2nd Road, Yu Zhong District, Chongqing, 400014 People’s Republic of China; 2 China International Science and Technology Cooperation Base of Child Development and Critical Disorders, Chongqing, China; 3 Chongqing Key Laboratory of Pediatrics, Chongqing, China

**Keywords:** Intestinal dysbacteriosis, Bronchopulmonary dysplasia, Macrophages, Gut microbiota, Antibiotic, Neonate

## Abstract

**Background:**

Modification of the gut microbiota by antibiotics may influence the disease susceptibility and immunological responses. Infants in the neonatal intensive care unit (NICU) subjected to frequent antibiotics and oxygen therapies, which may give rise to local and systemic inflammatory reactions and progression of bronchopulmonary dysplasia (BPD). This study aimed to investigate the role of intestinal dysbacteriosis by antibiotic therapy before hyperoxia exposure in the progression of BPD.

**Methods:**

Mice had been exposed to hyperoxia (85% O_2_) since postnatal day 3 until day 16 for the BPD model establishment, treated with antibiotics from postnatal day 2 until day 8. Treated mice and appropriate controls were harvested on postnatal day 2 or 10 for 16S rRNA gene sequencing, or postnatal day 17 for assessment of alveolar morphometry and macrophages differentiation.

**Results:**

Antibiotic-induced intestinal dysbacteriosis before hyperoxia exposure gave rise to deterioration of BPD evidenced by reduced survival rates and alveolarization. Moreover, antibiotic-induced intestinal dysbacteriosis resulted in increased M1 macrophage maker (iNOS) and decreased M2 macrophage maker (Arg-1) levels in lung homogenates.

**Conclusion:**

Broad-spectrum antibiotic-induced intestinal dysbacteriosis may participate in BPD pathogenesis via alteration of the macrophage polarization status. Manipulating the gut microbiota may potentially intervene the therapy of BPD.

**Supplementary Information:**

The online version contains supplementary material available at 10.1186/s12967-021-02794-6.

## Introduction

As a multi-factorial chronic lung disease of preterm infants, Bronchopulmonary dysplasia (BPD) subjected to interrupted lung deterioration [1]. It most commonly contributed to long-term morbidity and mortality in effectively low birth body weight (ELBW) babies [2]. Therefore, here comes a necessity for the understanding of the mechanisms leading to BPD. Infants in the neonatal intensive care unit (NICU) frequently receive antibiotic therapy [3]. Exposure to antibiotics was one of the most critical factors altering the gut microbiota of neonates [4, 5]. Immunological homeostasis rely on the microbiota, the metabolites and components of microbiota influence how the host susceptible to many immunological-mediated diseases and disorders [6]. Intestinal dysbacteriosis may influence the pulmonary immunological response through the intestinal-pulmonary axis [7, 8]. However, the role of intestinal dysbacteriosis in the BPD development remains unclear. Hence, this study aimed to investigate the underlying mechanism and role of intestinal dysbacteriosis in BPD.

## Materials and methods

### Animals

Pregnant C57BL/6 J mice were bought from the Experimental Animal Center of Chongqing Medical University (Chongqing, China) and were allowed to deliver spontaneously. All animal experiments were carried out pursuant to the protocols approved by the Animal Care and Use Ethics Committee of the Chongqing Medical University (No. 2019389; Chongqing, China). All mice were under the environment where temperature and humidity were controlled (Additional files 1, 2).

### Antibiotics treatment (ABX) and BPD model establishment

Neonatal mice were randomly divided into 4 groups: the Saline/Air group; the ABX/Air group; the Saline/O_2_ group; and the ABX/O_2_ group; n = 25 each. We treated the neonatal C57BL/6 mice with broad-spectrum antibiotics from postnatal day (PN) 2 to PN8. Antibiotics treatment involved oral gavage of ampicillin (10 mg/kg; Sangon Biotech), vancomycin (5 mg/kg; Sangon Biotech), neomycin sulfate (10 mg/kg; Sangon Biotech) and metronidazole (10 mg/kg; Sangon Biotech) every 12 h [9–11]. The control mice were perfused with saline every 12 h. Separate therapeutic experiment for mice with antibiotics and with saline was carried out in different cages and the neonatal mice were fed by their mother.

**Fig. 1:**
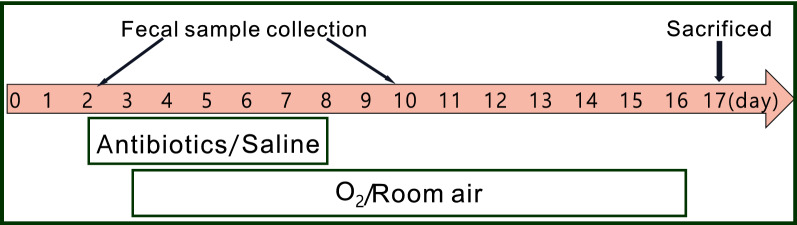
Treatment regime schematic for the bronchopulmonary dysplasia (BPD) model establishment and administration of antibiotics. The mouse model of BPD was conducted and treated with broad-spectrum antibiotics. The newborn C57BL/6 mice were treated with broad-spectrum antibiotics (ampicillin (10 mg/kg), vancomycin (5 mg/kg), neomycin sulfate (10 mg/kg) and metronidazole (10 mg/kg)) every 12 h by gavage from postnatal day (PN) 2 to PN8. The control mice were perfused with saline every 12 h from PN2 to PN8. In the case of the BPD model establishment, the newborn mice were exposed to hyperoxia (85% O_2_) from PN3 to PN16. The control mice were exposed to room air (21% O_2_) from PN3 to PN16

In the case of the BPD model establishment where mice received antibiotics or saline on PN2, hyperoxia exposure was initiated on PN3. The mouse modeling of BPD was performed pursuant to prior study [12]. Neonatal mice along with their mothers were exposed to hyperoxia (85% O_2_) or room air (21% O_2_) from PN3 to PN16 for 14 consecutive days (Fig. 1). Dams were rotated between hyperoxia and room air groups every 24 h to reduce oxygen toxicity and have free access to food and water. Fecal samples were collected at PN2 or PN10, lungs were collected at PN17 (Fig. 1).

### Histopathological analysis

The left lung of every mouse subjected to immersion in 4% paraformaldehyde, fastening in paraffin, and sectioning at 5 μm. Sections were stainted with hematoxylin and eosin (HE) and evaluated by light microscopy.

### Immunofluorescence

Lung tissue sections were submerged in the xylene and graded alcohols to deparaffinize and rehydrate, santigen retrieval was done using ethylenediaminetetraacetic acid (EDTA, pH 8.0). Sections blocking with blocking solution containing 10% donkey serum was performed at room temperature for 30 min. Then Sections incubation was performed with mouse anti-CD68 (1:200, Zenbio), rabbit anti-iNOS (1:200, Zenbio), rabbit anti-Arg-1 (1:200, Zenbio) at 4 °C overnight. The sections washing and incubation were performed with the secondary fluorescein Alexa Fluor 488 (1:400, green, Beyotime) and Cy3 (1:300, red, Beyotime) antibodies at roomtemperature for 50 min. Furthermore, nuclei was stained with 4′, 6-diamidino-2-phenylindole (DAPI, Leagene) at room temperature for 10 min. Finally, the sections washing and incubation were performed with AutoFluo Quencher (Beyotime) for 5 min and sections were viewed by fluorescent microscopy (Nikon, Japan).

### Western blot analysis.

Western blotting analysis was made to evaluate the expressions of iNOS and Arg-1 in the lung tissues. Lung tissues lysing in protein lysis buffer and protein concentration were evaluated with the Bradford approach. Lysates were separated by sodium dodecyl sulfate polyacrylamide gel electrophoresis (SDS-PAGE) and lysates transfer to polyvinylidene difluoride (PVDF) membranes (Millipore) were made. After 1 h membranes blocking with TBST buffer containing 5% non-fat dry for 1 h at room temperature, membranes incubation was performed with 1:800 anti-iNOS (Zenbio), 1:1000 anti-Arg-1 (Zenbio) and 1:1000 anti-β-actin (Zenbio) antibody overnight at 4 °C. After membranes washing with TBST (5 min × 3), 1 h membranes incubation was performed at room temperature with a secondary antibody (anti-rabbit IgG, 1:5000; Proteintech) diluted in TBST. Then immunoblots were visualized using ECL kit (Bio-Rad, USA).

### Fecal sample collection and assay of commensal bacteria in the intestine of neonatal mice

Due to the difficulty of gathering sufficient intestinal contents from neonates for 16 s sequencing, we chose to collect intestinal contents from 6 to 8 neonates per group [13]. We collected the intestinal contents as done previously [4]. QIAamp DNA stool Mini Kit (Qiagen) was applied for the extraction of bacterial DNA from the intestinal contents as instructed by the manufacturer [14]. Illumina MiSeq platform was applied for the sequencing of the 16S rRNA V3–V4 hypervariable region of bacteria according to protocols. The microbial community analysis was conducted as previously reported [15]. The community structure was analyzed by R package vegan 2.0 [16].

### Statistical analysis

Statistical analysis was made with SPSS version 22.0. Expression of quantitative data is denoted as mean ± standard deviation. In addition, unpaired two-tailed Student’s t-test or ANOVA or Wilcoxon signed-rank test was applied for the comparison of differences between groups. P < 0.05 was deemed significant in terms of statistics.

## Results

### Composition analysis of gut microbiota

**Fig. 2:**
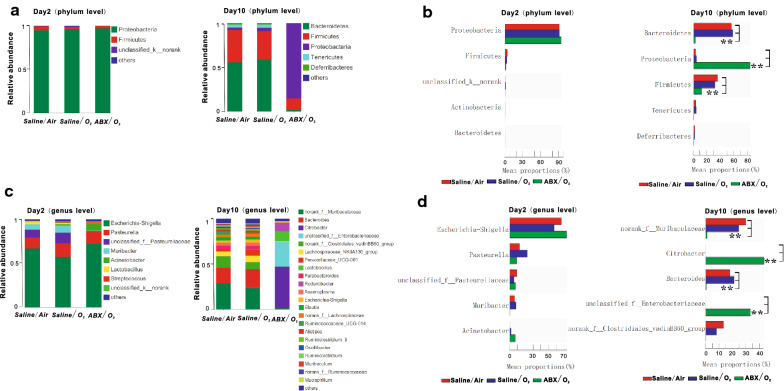
Antibiotic treatment changes the microbiota composition in neonatal mice. **a, b** The relative abundance of microbial communities at the phylum level on day 2 (before antibiotics treatment) and day 10 (after seven days treatment with antibiotics). **c, d** The relative abundance of microbial communities at the genus level on day 2 (before antibiotics treatment) and day 10 (after seven days treatment with antibiotics). Relative abundance > 1%, *P < 0.05. **P < 0.01

We collected fecal samples before the antibiotics treatment (Day 2), and after seven days of antibiotics therapy (Day 10). Colonization occurs gradually after birth. We found that neonatal mice were mainly colonized with *Proteobacteria* in the early life (Day 2), and then it was gradually replaced by *Firmicutes* and *Bacteroides* (Day 10) (Fig. 2)*.* After seven days of antibiotics therapy, *Proteobacteria* accounted for a higher proportion at the phylum level, whereas *Firmicutes* and *Bacteroidetes* accounted for a reduced proportion in the antibiotics treated mice than that in the saline treated mice (Fig. 2a, b, P < 0.01). At the genus level, the proportion of *Citrobacter* and *unclassified_f__Enterobacteriaceae* elevated, whereas the proportion of *Bacteroides* and *norank_f__Muribaculaceae* reduced in the antibiotics treated mice than that in the saline treated mice (Fig. 2c, d, P < 0.01).

### Effect of antibiotics treatment on survival and alveolarization in BPD mice

**Fig. 3:**
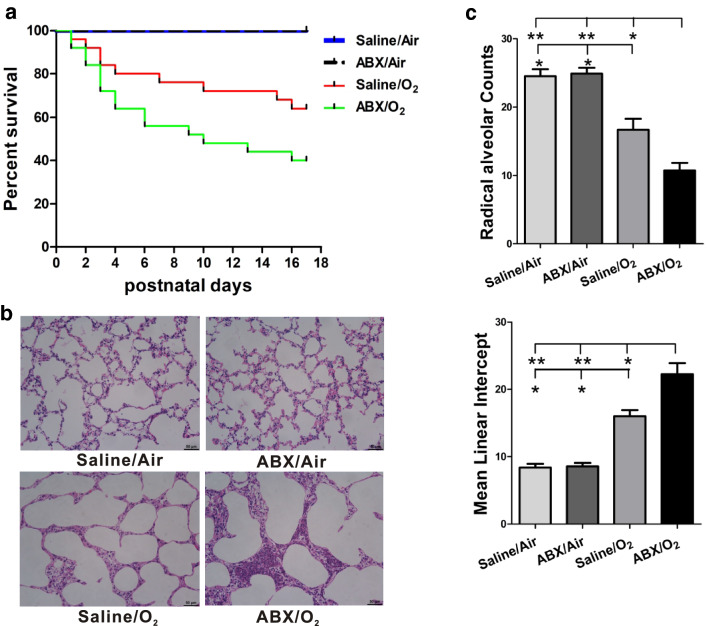
Effect of antibiotics treatment (ABX) on survival and alveolarization in BPD mice. **a** Survival of mice in the Saline/Air, ABX/Air, Saline/O_2_, ABX/O_2_ groups. The data represent 25 mice per group. **b** Representative H&E-stained sections of neonatal lungs in different groups. **c** Radial alveolar count (RAC) and mean linear intercept (MLI) of mice in different groups. Data were displayed as mean ± standard deviation. *P < 0.05, **P < 0.01. Results were analyzed using ANOVA

The survival rate of BPD mice reduced in the antibiotics treated mice than that in the saline treated mice (Fig. 3a, P < 0.05). Figure 3b, c showed that, the lungs of BPD mice had an elevated mean linear intercept (MLI) and decreased radial alveolar count (RAC) after exposed to hyperoxia. Moreover, lungs of mice in the ABX/O_2_ group had an elevated MLI and reduced RAC compared to the Saline/O_2_ group (Fig. 3b, c, P < 0.05).

### Effects of antibiotics treatment on polarization of macrophages in BPD mice

**Fig. 4:**
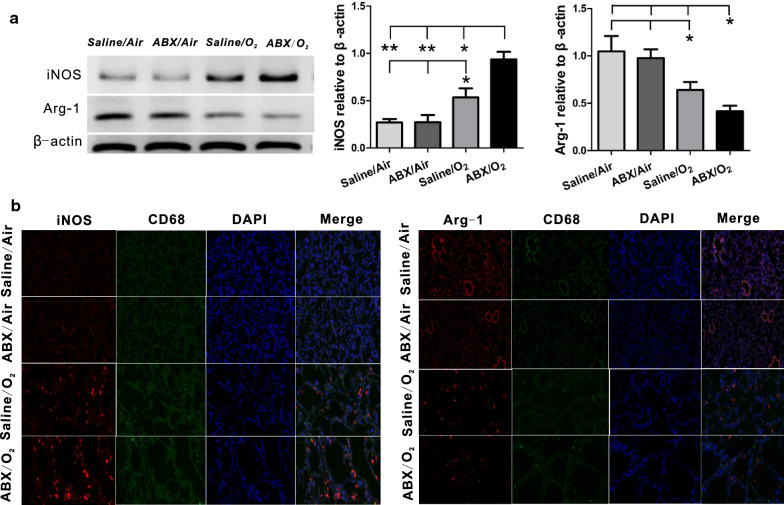
Effects of antibiotics treatment (ABX) on polarization of macrophages in BPD mice. **a **The iNOS and Arg-1 expressions were evaluated by western blot and relative gray intensity to β-actin was calculated. **b** Lung sections were stained by double immunofluorescence with anti-CD68 (macrophage marker, green), anti-Arg-1 (M2 marker, red) and anti-iNOS (M1 marker, red) antibodies. *P < 0.05, **P < 0.01. Results were analyzed using ANOVA

As the polarization of macrophages has potential relevance with pathogenesis of BPD [17], we observed whether the antibiotics treatment aggravated BPD via M1/M2 polarization pathways. The polarization of macrophages was identified by double immunofluorescence staining using anti-iNOS (M1) antibodies, anti-Arg-1 (M2) antibodies (Fig. 4b). The iNOS expression increased in BPD mice and then was upregulated when ABX was added (Fig. 4b). The Arg-1 expression decreased in BPD mice and then was inhibited when ABX was added (Fig. 4b). Then, the protein levels of iNOS and Arg-1 were detected by western blot. Consistent with the above results, iNOS increased in BPD mice and antibiotics treatment upregulated the expression of iNOS only when mice exposed to hyperoxia (Fig. 4a, P < 0.05). Arg-1 decreased in BPD mice and ABX inhibited Arg-1 expression only when mice exposed to hyperoxia (Fig. 4a, P < 0.05). Therefore, with the antibiotics treatment, the ratio of M1 macrophages increased and the ratio of M2 macrophage decreased in BPD mice, which indicates that ABX upregulated the ratio of M1/M2 macrophages as a pro-inflammatory factor in BPD mice.

## Discussion

In this study we found that antibiotic-induced intestinal dysbacteriosis prior to hyperoxia exposure increased the susceptibility of mouse BPD, evidenced by decreased RAC, increased MLI and increased mortality in the antibiotics treated BPD mice. These findings supported the previous hypothesis that the preterm infants who frequently received antibiotics therapy could develop more severe BPD [18].

BPD is the most common complicating disease of premature birth. Neonates with BPD usually subjected to respiratory sequelae [19, 20]. BPD remains a substantial challenge to the neonatologist. Therefore, in this study, we investigated the potential mechanisms of BPD through animal experiments.

Infants in NICU often receive antibiotics treatment. Antibiotics change the composition of gut microbiota. Gut microbiota composition is related to the immunological response. There are increasing evidences that support the concept of cross-talk between the gut microbiota and the lung. For example, Intestinal dysbacteriosis in mice results in abnormal airway allergic responses [21]. Microbiota depletion aggravates ventilator-induced lung injury [22]. These evidences supported that the gut microbiota may affect the lung mucosa by influencing the immunological response. But the participation of antibiotic-induced intestinal dysbacteriosis in the progression of BPD remains unclear. This study researched the effect of intestinal dysbacteriosis on hyperoxia exposure induced the BPD mouse model.

To investigate this interaction, neonates were administrated with a broad-spectrum antibiotic regimen for the induction of intestinal dysbacteriosis. The administration of antibiotics was showed to significantly change the composition of gut microbiota in neonatal mice. We found that the relative abundance of the phylum *Firmicutes*, *Bacteroidetes* and the genus *Bacteroides* and *norank_f__Muribaculaceae* was significantly decreased in the antibiotics treated mice. In contrast, the relative abundance of the phylum *Proteobacteria* and the genus *Citrobacter* and *unclassified_f__Enterobacteriaceae* was significantly increased in the antibiotics treated mice. The findings were consistent with the previous studies, which showed that antibiotics induced intestinal dysbacteriosis could cause decreased relative abundance of phylum *Bacteroidetes* and *Firmicutes* and increased relative abundance of *Proteobacteria* [23, 24].

According to the previous reports, macrophage polarization may play important roles in the development of BPD [25, 26]. When the microenvironment changes, activated macrophages can be M1- or M2-polarized [27]. Modulating the M1/M2 polarization status of macrophages can affect the severity of acute inflammatory conditions of the lung [25]. The M1-polarized macrophage can promote the production of some pro-inflammatory substances such as iNOS, which participate in inflammation and host defense [28, 29]. The M2-polarized macrophage is involved in tissue repair and tissue remodelling by secreting substances such as Arg-1 [30]. Some research highlighted the importance of Arg-1 in the lung development, as Arg-1 levels in the postnatal lung could be significantly increased during alveolarisation [25]. In this study, we found that antibiotics treatment promoted M1 marker iNOS expression and inhibited M2 marker Arg1 expression in the BPD mice. Hence, antibiotics treatment promoted the macrophages transformation towards pro-inflammatory M1 phenotype and inhibited the macrophages transformation towards anti-inflammatory M2 phenotype. So antibiotics treatment may promote inflammations and inhibit anti-inflammations resulting in the aggravation of BPD. Meanwhile, another study showed that after the composition of gut microbiota were changed and their metabolites were depleted during antibiotic administration, the supplementation of antibiotics with the metabolites could promote the macrophages transformation towards anti-inflammatory M2 phenotype [31]. Based on the above evidences, we suggested that antibiotics treatment may aggravate the mouse BPD via modulating the M1/M2 polarization status of macrophages.

There are limitations need to be considered. Firstly, there was a relatively high mortality in the experimental mice. The reason may be that experiments were conducted in neonatal mice subjected at 2–3 days of life. Secondly, antibiotics administration may affect the results of this study, the administration of antibiotics with fecal microbiota transplants (FMT) may help us to understand the role of microbiota in the development of BPD.

## Conclusion

In summary, our study showed that hyperoxia induced BPD was aggravated by antibiotic-induced intestinal dysbacteriosis in mice, possibly via alteration of the macrophage polarization status. The findings highlight a potentially role of intestinal dysbacteriosis in the BPD pathogenesis. Manipulating the gut microbiota may be a potential therapy of BPD.

## Supplementary Information

**Additional file 1:** The ethics documents for this study

**Additional file 2:** Translations of the ethics approvals.

## Data Availability

All data generated or analysed during this study are included in this published article.

## Abbreviations

BPD: Bronchopulmonary dysplasia
ELBW: Extremely low birth weight
PN: Postnatal day
NICU: Neonatal intensive care unit
ABX: Antibiotics treatment
HE: Hematoxylin and eosin
EDTA: Ethylenediaminetetraacetic acid
DAPI: 4′, 6-Diamidino-2-phenylindole
SDS-PAGE: Sodium dodecyl sulfate polyacrylamide gel electrophoresis
PVDF: Polyvinylidene difluoride
RAC: Radial alveolar count
MLI: Mean linear intercept
FMT: Fecal microbiota transplants
