# Supplementary figures and images for: Effect of antibiotic-induced intestinal dysbacteriosis on bronchopulmonary dysplasia and related mechanisms
Source: J Transl Med. 2021 Apr 16;19:155. doi: 10.1186/s12967-021-02794-6 (PMC8054697; doi:10.1186/s12967-021-02794-6)

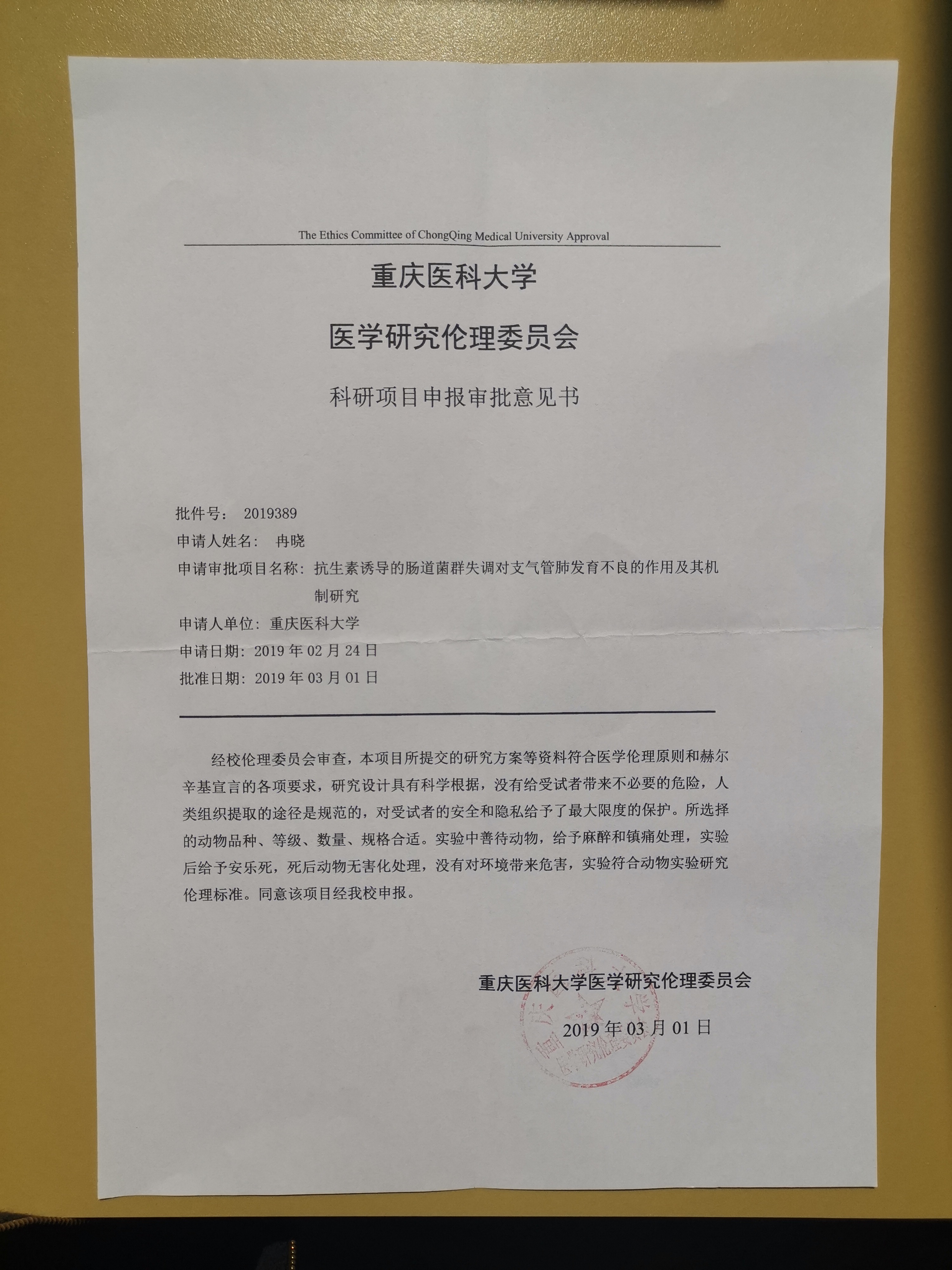

Supplement: Supplementary file 1 — Additional file 1: The ethics documents for this study [file 12967_2021_2794_MOESM1_ESM.jpg]

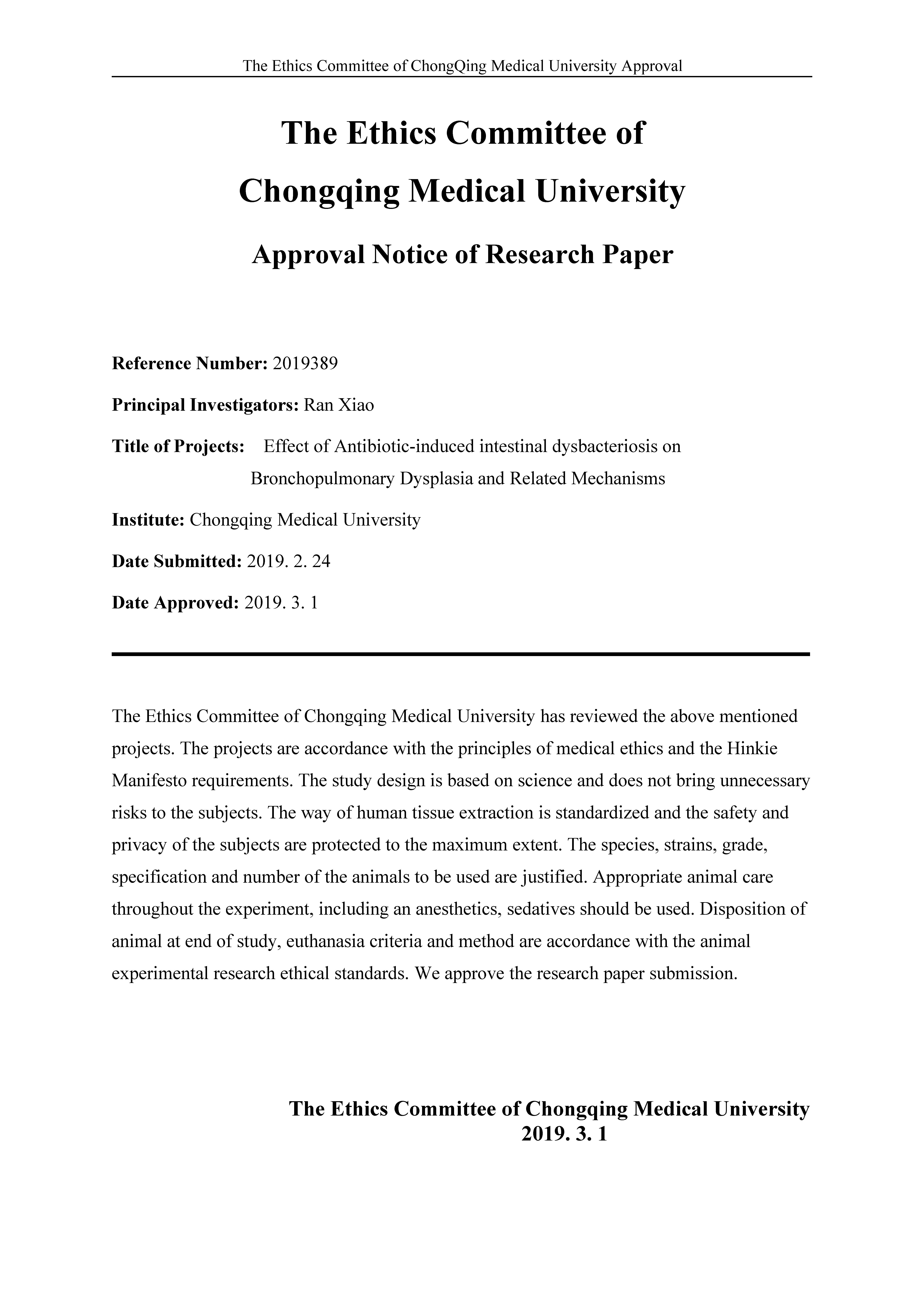

Supplement: Supplementary file 2 — Additional file 2: Translations of the ethics approvals. [file 12967_2021_2794_MOESM2_ESM.jpg]
